# Lectin-Like Molecules of *Lactobacillus rhamnosus* GG Inhibit Pathogenic *Escherichia coli* and *Salmonella* Biofilm Formation

**DOI:** 10.1371/journal.pone.0161337

**Published:** 2016-08-18

**Authors:** Mariya I. Petrova, Nicole C. E. Imholz, Tine L. A. Verhoeven, Jan Balzarini, Els J. M. Van Damme, Dominique Schols, Jos Vanderleyden, Sarah Lebeer

**Affiliations:** 1 KU Leuven, Centre of Microbial and Plant Genetics, Kasteelpark Arenberg 20, box 2460, B-3001, Leuven, Belgium; 2 University of Antwerp, Department of Bioscience Engineering, Groenenborgerlaan 171, B-2020, Antwerp, Belgium; 3 KU Leuven, Rega Institute for Medical Research, Minderbroedersstraat 10, B-3000, Leuven, Belgium; 4 Ghent University, Laboratory of Biochemistry and Glycobiology, Department of Molecular Biotechnology, Coupure Links 653, B-9000, Ghent, Belgium; University of Kansas Medical Center, UNITED STATES

## Abstract

**Objectives:**

Increased antibiotic resistance has catalyzed the research on new antibacterial molecules and alternative strategies, such as the application of beneficial bacteria. Since lectin molecules have unique sugar-recognizing capacities, and pathogens are often decorated with sugars that affect their survival and infectivity, we explored whether lectins from the probiotic strain *Lactobacillus rhamnosus* GG have antipathogenic properties.

**Methods:**

The genome sequence of *L*. *rhamnosus* GG was screened for the presence of lectin-like proteins. Two genes, *LGG_RS02780* and *LGG_RS02750*, encoding for polypeptides with an *N*-terminal conserved L-type lectin domain were detected and designated Llp1 (lectin-like protein 1) and Llp2. The capacity of Llp1 and Llp2 to inhibit biofilm formation of various pathogens was investigated. Sugar specificity was determined by Sepharose beads assays and glycan array screening.

**Results:**

The isolated lectin domains of Llp1 and Llp2 possess pronounced inhibitory activity against biofilm formation by various pathogens, including clinical *Salmonella* species and uropathogenic *E*. *coli*, with Llp2 being more active than Llp1. In addition, sugar binding assays with Llp1 and Llp2 indicate specificity for complex glycans. Both proteins are also involved in the adhesion capacity of *L*. *rhamnosus* GG to gastrointestinal and vaginal epithelial cells.

**Conclusions:**

Lectins isolated from or expressed by beneficial lactobacilli could be considered promising bio-active ingredients for improved prophylaxis of urogenital and gastrointestinal infections.

## Introduction

Antibiotic resistance is a growing issue representing a major challenge for healthcare. The inadequate and inappropriate use of antibiotics in the last decades has led to an increasing incidence of multidrug-resistant bacteria both in hospitals and the community [1]. The situation becomes even more severe taking the capacity of bacterial pathogens to form biofilms on biotic and abiotic surfaces into account, thereby limiting the effect of the available antibiotics [2,3]. Therefore, novel antibacterial agents with the capacity to inhibit bacterial biofilms are important for the treatment of bacterial infections. A promising class of antimicrobial molecules is the family of lectins [4]. Lectins are defined as carbohydrate binding proteins without catalytic activity that are prevalent in all organisms. They often have important functions in cell signaling and cellular interactions [5]. Currently, lectins are especially explored as antiviral agents, since the discovery that infection of heavily glycosylated viruses, such as human immunodeficiency virus (HIV- 1) and hepatitis C virus (HCV), can efficiently be blocked by a variety of lectins in cell and animal models [4].

Similar to plants and animals, bacteria can also express lectins on their surface, but reports on their characterization, and especially antipathogenic potential, are very scarce. In the present study, we aimed at the characterization of L-type lectin-like proteins with antimicrobial potential in the model probiotic strain *Lactobacillus rhamnosus* GG [6]. Genome mining of *L*. *rhamnosus* GG resulted in the identification of two genes encoding a putative L-type lectin-like protein. Knock-out mutant analysis and characterization of the purified lectin domains revealed that these novel bacterial lectins exert a unique pathogenic biofilm inhibitory activity and promote adhesion to host cells, suggesting that they might be suitable for future strategies to topically fight bacterial pathogens.

## Material and Methods

### Bacterial strains, plasmids and growth conditions

The bacterial strains and plasmids used in this study are listed in Table 1. *L*. *rhamnosus* GG wild type, the corresponding mutants and the different *Lactobacillus* strains were routinely grown non-shaking in de Man Rogosa Sharpe (MRS) medium (Difco) at 37°C. Alternatively, Bacto Lactobacilli AOAC medium (Difco) was used for the analysis of biofilm formation as previously described [7]. *Escherichia coli* strains, *Pseudomonas aeruginosa* PA14, *Staphylococcus aureus* strain Rosenbach and strain SH1000, and *Salmonella enterica* serovar Typhimurium ATCC14028 and all human isolates were grown in Luria Bertani (LB) medium with aeration at 37°C. If required, antibiotics were used at following concentrations: 10 μg/ml tetracycline, 100 μg/ml ampicillin, 50 μg/ml kanamycin, 10μg/ml chloramphenicol, 5 μg/ml (for *L*. *rhamnosus* GG) or 130 μg/ml (for *E*. *coli*) erythromycin. During biofilm and bioscreen experiments, *E*. *coli* UTI89, *Salmonella* strains and *P*. *aeruginosa* PA14 were grown in 1/20 diluted tryptic soy broth (TSB, Gibco) and *S*. *aureus* Rosenbach and SH1000 in undiluted TSB.

**Table 1:** Strains and plasmids used in this study.

Strain/plasmid	Relevant genotype/description	Reference or source
***E*. *coli* strains**		
*E*. *coli* UTI89	Wild type, clinical isolate	[8]
*E*. *coli* K12 GM2163	F^-^ *ara-*14 *leu*B6 *fhu*A31 *lac*Y1 *tsx*78 *gln*V44 *gal*K2 *gal*T22 *mcr*A *dcm-*6 hisG4 *rfb*D1 *rps*136(Str^R^) *dam*13: : *Tn*9 (Cam^R^*) xyl*A5 *mtl-*1 *thi-*1 *mcr*B1 *hsd*^*R2*^	[9]
Top10F’	F’ (*lacI*^q^, Tn^r^) *mcr*A Δ(*mrr-hsd*RMS-*mcr*BC) Φ80*Lac*ZΔM15 Δ*lac*X74 *deo*R *rec*A1 *ara*D139 Δ(*ara-leu*)7697 *gal*U *gal*K *rps*L(St^r^) *end*A1 *nup*G	Invitrogen
*E*. *coli* BL21 (DE3)	*E*. *coli* B F- *dcm ompT hsdS(r*_*B*_^*-*^ *m*_*B*_^*-*^*) gal λ* (DE3)	Invitrogen
CMPG10708	*E*. *coli* BL21 (DE3) carrying the overexpression plasmid pCMPG10708 for secretion of N-His6 tagged Llp1 protein of *L*. *rhamnosus* GG, *Km*^*R*^	This study
CMPG10709	*E*. *coli* BL21 (DE3) carrying the overexpression plasmid pCMPG10709 for secretion of N-His6 tagged Llp2 protein of *L*. *rhamnosus* GG, *Km*^*R*^	This study
CMPG10712	*E*. *coli* BL21 (DE3) carrying the overexpression plasmid pCMPG10712 for secretion of N-His6 tagged Llp1 lectin-like domain from *L*. *rhamnosus* GG, *Km*^*R*^	This study
CMPG10713	*E*. *coli* BL21 (DE3) carrying the overexpression plasmid pCMPG10713 for secretion of N-His6 tagged Llp2 lectin-like domain from *L*. *rhamnosus* GG, *Km*^*R*^	This study
***L*. *rhamnosus* GG strains**		
Wild type	Human isolate	ATCC 53103 [10]
CMPG10701	*llp1* knock-out mutant of *L*. *rhamnosus* GG; *llp1*::*Tet*^*R*^,	This study
CMPG10706	*llp2* knock-out mutant of *L*. *rhamnosus* GG; *llp2*::*Ery*^*R*^	This study
CMPG10707	*llp1-llp2* double knock-out mutant of *L*. *rhamnosus* GG *llp1*::*Tet*^*R*^; *llp2*::*Ery*^*R*^	This study
CMPG10702	CMPG10701 complemented by electroporation of pCMPG10702 containing *llp1* gene.	This study
CMPG10715	CMPG10706 complemented by electroporation of pCMPG10715 containing *llp2* gene	This study
CMPG10773	CMPG10707 complemented by electroporation of pCMPG10715 containing the *llp2* gene ligated behind the *dlt* promotor	This study
***Lactobacillus* strains**		
*L*. *reuteri* RC-14 ATCC 55845	Wild-type, female urethra isolate	[11,12]
*L*. *crispatus* NCIMB 4505	Wild-type, human vaginal isolate	[13]
*L*. *jensenii* ATCC 25258	Wild-type, human vaginal isolate	[14]
*L*. *gasseri* ATCC 33323	Wild-type, human isolate	[14]
*L*. *plantarum* CMPG5300	Wild-type, human vaginal isolate	[15]
*L*. *rhamnosus* GR- 1 ATCC 5582	Wild-type, female urethra isolate	[11,12]
***Salmonella* strains**		
*Salmonella enterica* serovar Typhimurium ATCC 14028	Wild type, isolated from chicken tissue	ATCC [16]
*S*. Typhimurium ATCC 14028 carrying pFPV25.1	Mutant constitutively expressing GFP	[17]
*S*. *enterica* serovar Typhimurium SL1344	Wild type, *xyl his*G *rps*L; virulent; *Sm*^*R*^	[18]
*S*. *typhimurium* SGSC2196	Wild type, human isolate- SARA* collection	[19]
*S*. *typhimurium* SGSC2199	Wild type, human isolate- SARA collection	[19]
*Salmonella saintpaul* SGSC 2209	Wild type, human isolate- SARA collection	[19]
*Salmonella heidelberg* SGSC2213	Wild type, human isolate- SARA collection	[19]
*Salmonella paratyphi B* SGSC2221	Wild type, human isolate- SARA collection	[19]
*S*. *paratyphi B* SGSC2228	Wild type, human isolate- SARA collection	[19]
*Salmonella anatum* SGSC2459	Wild type, human isolate- SARB* collection	[20]
*Salmonella infantis* SGSC2483	Wild type, human isolate- SARB collection	[20]
*Salmonella miami* SGSC2485	Wild type, human isolate- SARB collection	[20]
**Other strains**		
*S*. *aureus* SH1000	*rsbU* positive derivative of *S*. *aureus* 8325–4	[21]
*S*. *aureus* Rosenbach (ATCC 33591)	Wild type, clinical isolate	ATCC
*P*. *aeruginosa* PA14	Wild type, human isolate	[22]
**Plasmids**		
pFAJ5301	Cloning vector; pUC18 derivative; *Ery*^*R*^	[23]
pET28 (a+)	*Km*^*R*^, T7 lac, N and C-terminal His Tag	Novagen
pCMPG10205	pUC18 containing tetracycline resistant cassette from pGK13 in the *Bsp*HI site	[24]
pCMPG10208	pLAB1301 derivative driven by *dlt* promoter *Amp*^*R*^, *Ery*^*R*^	[25]
pCMPG10212	pLAB1301 derivative driven by *dlt* promotor *Amp*^*R*^, *Cm*^*R*^	[26]
pCMPG10701	pCMPG10205 derivative used to inactivate *llp1* gene by insertion of a *Tet*^*R*^ marker via double homologous recombination	This study
pCMPG10702	pCMPG10208 derivative containing the *llp1* gene (2040 bp) in the *XmaI/SacI* site *Amp*^*R*^, *Ery*^*R*^	This study
pCMPG10705	pFAJ5301 derivative used to inactivate the *llp2* gene by insertion of a *Ery*^R^ market via single homologous recombination	This study
pCMPG10708	pET 28a(+) derivative carrying the *llp1* gene in the *SalI/NotI* site *Km*^*R*^	This study
pCMPG10709	pET 28a(+) derivative carrying the *llp2* gene in the *SalI/NotI* site *Km*^*R*^	This study
pCMPG10712	pET 28a(+) derivative carrying the lectin-like domain of the *llp1* gene in the *SalI/NotI* site *Km*^*R*^	This study
pCMPG 10713	pET 28a(+) derivative carrying the lectin-like domain of *llp2* gene in the *SalI/NotI* site *Km*^*R*^	This study
pCMPG10715	pCMPG10212 derivative containing the *llp2* gene (2078 bp) in the *Xma/SacI* site *Amp*^*R*^, *Cm*^*R*^	This study

*SAlmonella Reference Collection A or B; *Ery*^*R*^- erythromycin resistance; *Tet*^*R*^- tetracycline resistance; *Km*^*R*^- kanamycin resistance, *Amp*^*R*^- ampicillin resistance; *Cm*^*R*^- chloramphenicol resistance.

### DNA manipulations

Routine molecular biology techniques were performed as described before [27]. PCR primers used in this study (Table 2) were purchased from Integrated DNA Technologies (IDT) (Belgium). Enzymes for molecular biology were purchased from New England Biolabs (Belgium) and used according to the suppliers instructions. Plasmid DNA preparation from *E*. *coli* was performed using QIAGEN miniprep kits.

**Table 2:** List of primers used in this study.

Primer	Sequence (5´-3´)	Restriction site	Remarks
M13 Universe	CGACGTTGTAAAACGACGGCCAGT	/	Forward primer to check insertion in multiple cloning site of pCMPG10205
M13 Reverse	CAGGAAACAGCTATGAC	/	Reverse primer to check insertion in multiple cloning site of pCMPG10205
Pro4655	ATCCCGGGAGCCAGCGCGGTTAGAAGCC	*SmaI*	Forward primer HR1 *llp1* gene LGG
Pro4656	ATCCCGGGATCGACGCCGCTTCGCCTAC	*SmaI*	Reverse primer HR1 *llp1* gene LGG
Pro4658	ATGCGGCCGCCGGAACGCTCAGTGGCGACG	*NotI*	Forward primer HR2 *llp1* gene LGG
Pro4659	ATGTCGACTACACGCTGCTGCTGCCTCTCGCAC	*SalI*	Reverse primer HR2 *llp1* gene LGG
Pro5112	GCAGATGCTGCAAGCGCGAC	*/*	Forward primer to check *llp1* replacement
Pro5113	TGCAACATGTGCAACGCCGCTTA	*/*	Reverse primer to check *llp1* replacement
Pro5726	ATCCCGGGGCACCGGTTCACGCTCACCA	*XmaI*	Forward primer complementation *llp1* gene
Pro5727	ATGAGCTCTGCAACATGTGCAACGCCGC	*SacI*	Reverse primer complementation *llp1* gene
Pro5841	ATAAGCTTTGGGGCGGCGCAGATGGGAG	*HindIII*	Forward primer *llp2* gene LGG
Pro5842	ATGAATTCCCCCGTTTGCGTTGCCGTTG	*EcoRI*	Reverse primer *llp2* gene LGG
Pro5880	CACCGTCGACCGAAGAAGAAATATTCA	*Sal*I	Forward primer for full length *llp1* gene for pET28 a(+)
Pro5881	ACTGGCGGCCGCTTAAGGCATAGGAGTAG	*Not*I	Reverse primer for full length *llp1* gene for pET28 a(+)
Pro5882	CACCGTCGACCGAAGAAGTGCGGCTACCT	*Sal*I	Forward primer for full length *llp2* gene for pET28 a(+)
Pro5883	ACTGGCGGCCGCTCACTGAAGAGCGTT	*Not*I	Reverse primer for full length *llp2* gene for pET28 a(+)
Pro6186	ATCCCGGGGCAAACCGGTGATGCCGTGC	*SmaI*	Forward primer complementation *llp2*
Pro6187	ATCCCGGGAGCTGAACCCCTTTTTCAACTC	*SmaI*	Reverse primer complementation *llp2*
S&P-00517	ATGTCGACAAGGGTGGCCGTCATCGTCAGG	*SalI*	Forward primer upstream of lectin-like domain of *llp1* gene
S&P-00518	ATGCGGCCGCTTAATCTTCTACCTTCAAATGCGTG	*NotI*	Reverse primer downstream of lectin-like domain of *llp1* gene
S&P-00620	ATGTCGACAACCAAAATGGCCAAGCCC	*SalI*	Forward primer upstream of lectin-like domain of *llp2* gene
S&P-00621	ATGCGGCCGCTTATACGGCGCCTTTAATTTGATT	*NotI*	Reverse primer downstream of lectin-like domain of *llp2* gene
S&P-0044	TGGCAGCAGCCAACTCAGCTT	/	Reverse primer for MCS of pET28 a(+)
S&P-0045	TATAGGCGCCAGCAACCGCA	/	Forward primer for MCS of pET28 a(+)

### Identification and sequence analysis of the *L*. *rhamnosus* GG *llp1* and *llp2* genes

The genome sequence of *L*. *rhamnosus* GG was mined for the presence of putative lectin-like proteins by BLAST using the mannose-specific adhesin (Msa) protein of *L*. *plantarum* WCFS1 [28]. This resulted in the identification of genomic regions encoding two putative lectin-like proteins of which the putative gene sequence was designated as *llp1* (cfr. *LGG_RS02780*) and *llp2* (*LGG_RS02750*), respectively.

### Construction of knock-out mutants in the lectin-like proteins in *L*. *rhamnosus* GG

To determine the role of the *LGG_RS02780* gene, a corresponding knock-out mutant termed CMPG10701 (Table 1) was constructed by double homologous recombination as previously described [7]. Subsequently, a knock-out mutant in *LGG_RS02750* was constructed by PCR using primers Pro5841 and Pro5842 and subsequent cloning the amplicon into plasmid pFAJ5301 resulting into plasmid pCMPG10705. The resulting plasmid containing *LGG_RS02750* gene was isolated and transferred to highly competent *L*. *rhamnosus* GG wild type by electroporation as described above. Plasmid insertion into the *L*. *rhamnosus* GG genome was checked by PCR using primers Pro5610- M13 and Pro5611- M13. A putative knock-out mutant of *LGG_RS02750* gene was selected by its resistance to erythromycin and confirmed by PCR. Finally, a double mutant was constructed by transferring the plasmid pCMPG10705 into the *llp1* mutant CMPG10701 strain by electroporation, resulting in the double mutant *llp1-llp2* strain CMPG10707, which was selected by its ability to grow in medium containing erythromycin and confirmed by PCR.

### Construction of overexpression constructs of the lectin-like proteins in *E*. *coli* BL21 (DE3)

For heterologous expression of Llp1 and Llp2 proteins in *E*. *coli*, the pET 28 a(+) system (Novagen) was used. The *LGG_RS02780* and *LGG_RS02750* genes from *L*. *rhamnosus* GG wild type were amplified by PCR using the corresponding primers listed in Table 2. The *LGG_RS02780* and *LGG_RS02750* genes were cloned into the pET-28 a(+) vector (Novagen) resulting in plasmids pCMPG10708 and pCMPG10709, respectively. The plasmids were then transformed in competent *E*. *coli* strain BL21 (DE3) cells resulting into strain CMPG10708 and CMPG10709. In addition, the L-type lectin domains of Llp1 and Llp2 were also successfully amplified and cloned into pET-28 a(+) resulting in plasmids pCMPG10712 and pCMPG10713 for the L-type lectin domain from Llp1 and Llp2, respectively. pCMPG10712 and pCMPG10713 were successfully transformed to *E*. *coli* BL21 (DE3) and designated CMPG10712 and CMPG10713 respectively.

### Production of recombinant lectins and lectin domains and their purification

The recombinant *E*. *coli* BL21 (DE3) cells expressing the full length lectins or the corresponding lectin domains of LGG_RS02780 and LGG_RS02750 (CMPG10708, CMPG10709, CMPG10712 and CMPG10713) were grown overnight in LB medium with 50 μg/ml kanamycin. The production of recombinant protein was induced with 1 mM isopropyl β-D-thiogalactopyranoside (IPTG) and the cultures were incubated at 25°C with shaking until an OD of 0.8 to 1 was reached. The pellets were suspended in non-denaturing lysis buffer (NaH_2_PO_4_ 50 mM, NaCl 300mM, imidazole 20 mM) and incubated for 30 minutes at room temperature while swirling and sonicated to release the soluble recombinant lectins from the cells. The full length lectins or the corresponding lectin domains were purified from the cell lysate using affinity chromatography with a HisTrap^™^ HP column (GE Healthcare). The bound lectin (domain) was eluted using an elution buffer (NaH_2_PO_4_ 50 mM, NaCl 300mM, imidazole 250mM) and further purified by using size exclusion chromatography. Hereto the sample was applied on a Highload^™^ 16/60 column packed with a matrix of Superdex^™^ prep grade (GE Healthcare). Fractions containing the lectin (domain) were collected and analyzed using SDS-PAGE.

### SDS-PAGE and Western blot

To verify the expression of recombinant proteins, as well as the presence of pure lectin (domain) after purification steps, each fraction was separated by SDS-PAGE using Bolt 12% Bis-Tris Plus gels (Life sciences). The gels were run submerged in morpholinepropanesulfonic acid (MOPS) buffer for 45 minutes at 400 mA and 200 V. Hereafter, the gels were used for a Western blot or stained with Coomassie Brilliant Blue R-250 (Bio Rad) or Sypro^®^ Ruby protein gel stain (Invitrogen).

### Bacterial growth assays in suspension

The antimicrobial effect of the lectins on pathogenic growth was assessed by using 100-well microtiter plates (Honeycomb, Oy Growth Curves Ab Ltd.) (Bioscreen) as previously described [29]. Overnight cultures of *E*. *coli* UTI89 and *S*. Typhimurium ATCC14028 were 200-fold diluted in 1/20 TSB and 200 μl was added to sterile wells of 100-well microtiter plates (Honeycomb, Oy Growth Curves Ab Ltd). The purified lectin domains were added at concentration of 200 μg/ml. The bacteria were incubated for 3 days at 37°C with agitation in a Bioscreen (Oy Growth Curves Ab Ltd.), which measured the OD at 600 nm every 10 minutes. Each strain and lectin domain was tested in triplicate.

### Antimicrobial assays for pathogens grown in biofilms

Biofilm formation assays on static pegs were performed as previously described with minor modifications [30]. Hereto, *E*.*coli* K12, *E*. *coli* UTI98, *S*. *aureus* SH1000, *S*. *aureus* Rosenbach, *P*. *aeruginosa* PA14, different *Salmonella* strains and different *Lactobacillus* strains were grown on polystyrene pegs in the presence of purified full length lectins or lectin domains at a final concentration of 50 or 200 μg/ml. *E*. *coli* UTI98 and *S*. Typhimurium ATCC14028 were also grown in biofilms when adding different lectins concentrations, namely 200μl/ml, 150μl/ml, 100μl/ml, 50μl/ml, 40μl/ml, 30μl/ml, 20μl/ml, 10μl/ml, 5μl/ml, 1μl/ml, 500ng/ml, 250ng/ml. After 72 hours of growth, the biofilm formation was quantified by staining with crystal violet (0.1 w/v% in 5% methanol, 5% isopropanol and 90% PBS). For each strain and lectin domain, the experiment was performed at least three times with 8 technical repeats.

The total cell count of biofilms was also determined as previously described [17]. Briefly, *S*. Typhimurium ATCC14028 and *E*. *coli* UTI89 were allowed to form biofilms at the bottom of polystyrene wells of 12-well culture plates (Cellstar^®^). The lectin domains were added at 50 μg/ml. After incubation for 48h at 25°C or 37°C for *S*. Typhimurium ATCC14028 and *E*. *coli* UTI89, respectively, the biofilms were detached from the bottom of the wells using scrapers (Greiner bio-one) and pushed through a needle (25G, 0.5 x 16 mm, Terumo) to separate cellular aggregates. The dissolved biofilms were serially diluted in PBS and plated on LB. For each strain, the experiments were performed at least three times with three technical repeats.

For the visualization of *S*. Typhimurium biofilms, *S*. Typhimurium ATCC14028 carrying the pFPV25.1 plasmid was used, which constitutively expresses the *gfp*mut3 gene [17]. Microscopic epifluorescence imaging was performed using a Zeiss Axio Imager Z1 microscope with an EC Plan Neofluar (X40 magnification/0.3 numerical aperture) objective (excitation 488 nm, emission 511 nm). Pictures were acquired with an AxioCam MRm and the AxioVision software. Alternatively, wild type *S*. Typhimurium ATCC14028 or *E*. *coli* UTI89 and FITC-labeled lectin domains were used to visualize the biofilms.

### Pull-down carbohydrate binding assay using sepharose beads

Sepharose^®^ 6B beads (Sigma-Aldrich) were coated with 20% D-glucose, D-mannose, D-fucose, GlcNAc and mannan of *S*. *cerevisiae* as previously described with little modification [31,32]. For the sugar-binding assay, 25 μl of each functionalized bead was washed with binding buffer (25mM MES, 25mM NaCl and 1% polyvinylalcohol) as previously described [31]. Briefly, 1 ml of binding buffer containing 50 μg/ml of the purified lectin domain was added to each bead. Hereafter, the mixture was incubated at 4°C for 2 h. The beads were washed twice with 1 ml of wash buffer and bound lectin domains were eluted by boiling the beads in SDS-PAGE loading buffer (Fermentas, Life Sciences) for 10 min at 95°C. The bound lectin domains were resolved by SDS-PAGE through 12% polyacrylamide gels (Life Sciences), which were stained with Sypro^®^ Ruby protein gel stain (Invitrogen) and scanned by using the Typhoon scanner (GE Healthcare Life Sciences).

### Glycan array analysis

The mammalian glycan array version 5.2 was used to explore the carbohydrate binding specificity of the lectin domain of Llp1 and Llp2. The array consists of 609 glycan targets of natural and synthetic mammalian glycans with amino linkers and it is printed onto N-hydroxysuccinimide (NHS)-activated glass microscope slides (SCHOTT Nexterion), forming covalent amide linkages. The purified lectin domains of Llp1 and Llp2 were labeled with FITC by using FluoReporter^®^ FITC Protein Labeling Kit (Life Technologies) according to the producer’s manual. 200 μg/ml of FITC labeled protein was used to analyze the carbohydrate binding activity. The experiment was performed by the Consortium for Functional Glycomics (CFG, www.functionalglycomics.org).

### *In vitro* adhesion assay to a human epithelial cell lines

Adhesion assays using the Caco2 (ATCC HTB- 37TM) and VK2/E6E7 (ATCC CRL-2616^™^) cell lines were performed as previously described [33,34]. Alternatively, an immunofluorescence assay was performed as previously described [35] with minor modifications. Briefly, FITC labeled lectin domains were suspended in the DMEM medium in the absence of serum and antibiotics, and incubated for 1 h with the monolayer of Caco2 and VK2/E6E7 cells grown on the 13-mm coverslips. After incubation, cells were sequentially washed three times with PBS, and fixed with 4% paraformaldehyde for 10 min. Slides were examined with a Zeiss Axio Imager Z1 microscope with an EC Plan Neofluar (X40 magnification/0.3 numerical aperture) objective (excitation 488 nm, emission 511 nm). Pictures were acquired with an AxioCam MRm monochrome digital camera.

### Statistical analysis

To determine significant differences the unequal variance t-test was used. A P-value below 0.05 was considered as statistically significant

## Results

### The *LGG_RS02780* and *LGG_RS02750* genes encode Lectin-like protein 1 (Llp1) and Lectin-like protein 2 (Llp2)

**Fig 1:**
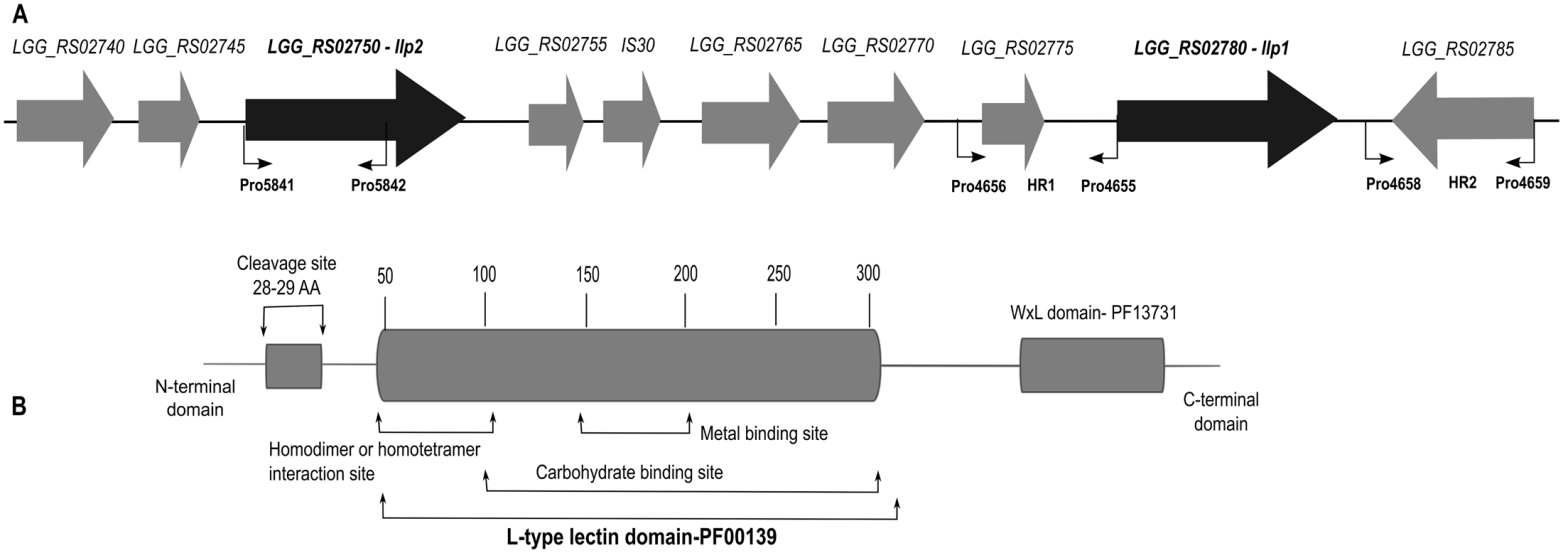
(A) The genomic region of the *LGG_RS02780* and *LGG_RS02750* genes with their surrounding genes. The two genes are separated by 6018 bp (5 genes). Of note, one gene located between *LGG_RS02780* and *LGG_RS02750*, i.e. *RS_02760*, belongs to the family of insertion elements *IS31* [36], suggesting a possible gene duplication event mediated by *IS31*. Gene *RS_02810* downstream of gene *LGG_RS02780* belongs also to the family of insertion elements (*IS32*) suggesting that probably the genome region of *LGG_RS02780* was inserted later in the genome of *L*. *rhamnosus* GG. The primer binding sites to amplify HR1 and HR2 for the construction of the CMPG10701 mutant as well as the 1000 bp region for the construction of the CMPG10706 mutant are indicated with arrows. (B) Putative protein domain organization of Llp1 and Llp2. The lectin-like domain (PF00139, clan CL0004) comprises ca. 250 amino acid residues and is predicted to contain three sites: (1) one responsible for specific carbohydrate recognition; (2) a metal-binding site and (3) the homodimer or homotetramer interaction site. The cleavage site in the *N*-terminal domain required for removal of the signal leader peptide and export of the protein out the bacterial cells is shown. The *C*-terminal WxL domain (PF13731) putatively responsible for anchoring the protein at the cell wall is depicted.

To identify genes encoding putative lectin-like proteins, the genome sequence of *L*. *rhamnosus* GG [10] was screened for the presence of open reading frames (ORFs) containing a lectin Legume (L)-type domain (PF00139). Two genomic regions encoding two putative cell wall proteins, i.e. *LGG_RS02780* and *LGG_RS02750* were identified (Fig 1A).

The *LGG_RS02780* and *LGG_RS02750* genes are 2041 bp and 2078 bp long, respectively, encoding polypeptides of 679 and 688 amino acid residues with a similar predicted protein domain organization (Fig 1B). The two proteins contain two conserved protein domains, i.e. an N-terminal Legume-type (L-type) lectin domain (PF00139) and a C-terminal WxL anchoring domain (PF13731). Because of the lectin domain, these gene sequences were annotated as *llp1* and *llp2*, encoding the putative lectin-like protein 1 and 2 (Llp1 and Llp2), respectively. The L-type lectin domain of Llp1 and Llp2 represents approximately 250 amino acid residues and their amino acid sequences show only 35% sequence similarity, suggesting that Llp1 and Llp2 result from a gene duplication event followed by diversification. Sequence comparisons with sequences in the NCBI protein database revealed a number of significant matches with proteins from lactobacilli. Unfortunately, all these proteins remain functionally uncharacterized, except for Msa from *L*. *plantarum* WCFS1 [28], which shows 22% and 16% sequence identity to Llp1 and Llp2, respectively.

### Llp1 and Llp2 inhibit biofilm formation of the key gastrointestinal pathogen *Salmonella enterica* serovar Typhimurium ATCC14028

To determine the antimicrobial activity of Llp1 and Llp2, their sequences were overexpressed in *E*. *coli* BL21 DE3. Despite numerous attempts, we succeeded to purify the full length Llp1 and Llp2 only in very low concentrations. Fortunately, overexpression of only the predicted L-type domains from Llp1 and Llp2 resulted in much higher yields and allowed us to purify the recombinant lectin domain from the soluble protein fraction of *E*. *coli* BL21 DE3 cells in sufficient amounts.

**Fig 2:**
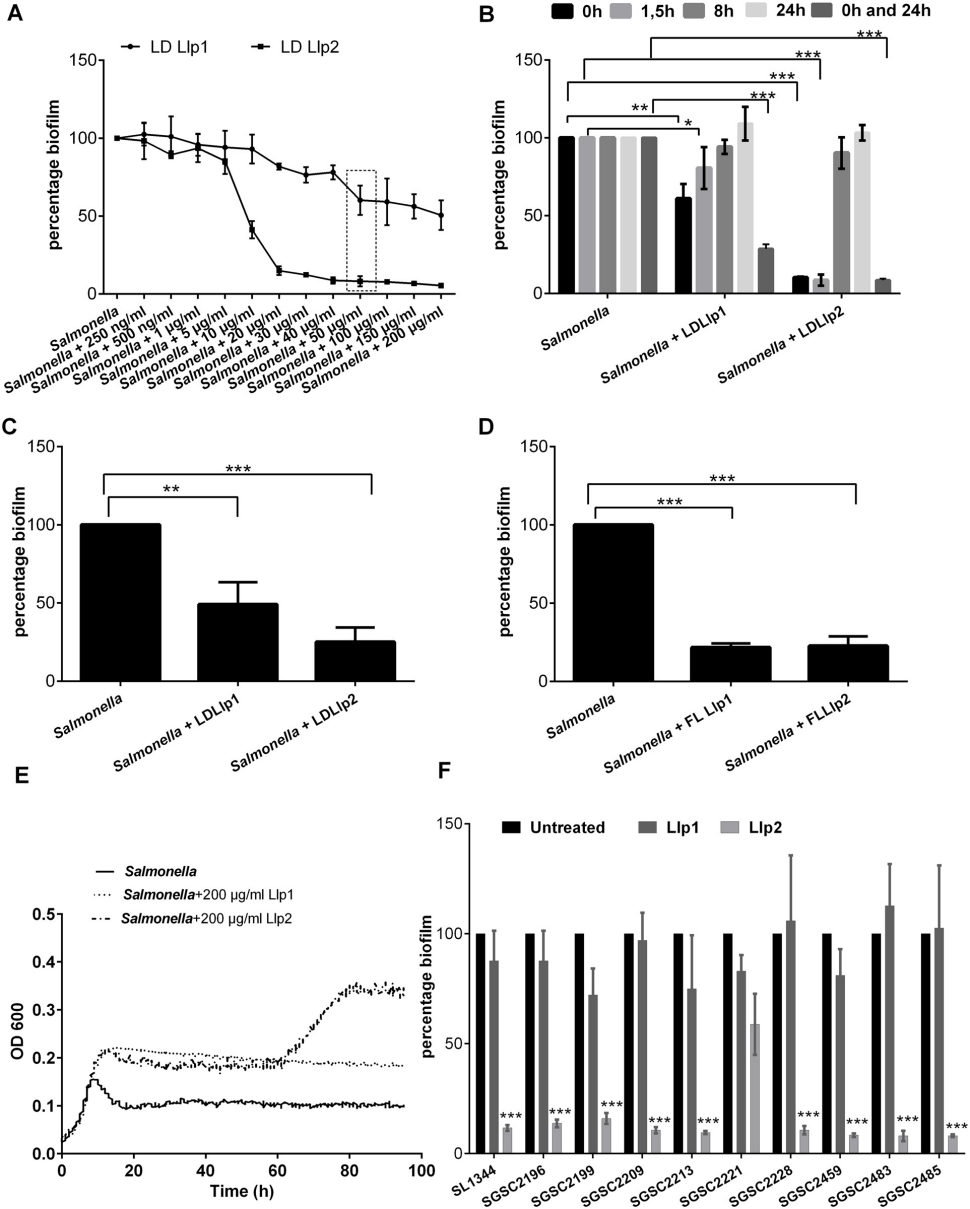
Antibiofilm activity of Llp1 and Llp2 against *S*. Typhimurium ATCC14028. (A) Effect of the lectin domains (LD) of Llp1 and Llp2 on *S*. Typhimurium ATCC14028 biofilms added at different concentrations at the start of the biofilm formation. The lowest concentration in which both of the lectins showed significant reduction in the biofilm is indicated with a shaded bow. (B) Effect of LD on *S*. Typhimurium ATCC14028 biofilms added after 0, 1.5, 8h, 24 h and after 0 and 24 h with fresh medium to the biofilms at a concentration of 50 μg/ml. (C) Absolute CFU *S*. Typhimurium ATCC14028 biofilms grown for 48h with lectins added at zero-time point at a concentration of 50 μg/ml. Absolute CFU counts of the treatments was normalized towards the control, which was grown in medium without lectins. (D) Effect of full length (FL) lectin (50 μg/ml) on *S*. Typhimurium ATCC14028 biofilms added at zero-time point to the biofilms. (E) Growth of *S*. Typhimurium ATCC14028 in presence of lectin domain of Llp1 and Llp2 (200 μg/ml) in 1/20 TSB medium. (F) Effect of lectin domains (50 μg/ml) on various *Salmonella* biofilms added at zero-time point. The error bars represent standard deviation of three independent experiments. The dataset comparisons are considered significant (p < 0.05 indicated with one asterisk in the picture, p < 0.01 indicated with two asterisks in the picture or p< 0.001 indicated with three asterisks on the picture).

To monitor interactions between the putative lectins and pathogens, biofilm assays were performed. First, varying concentrations of recombinant lectins were tested. These lectins were added at the start of the static peg biofilm assay (Fig 2A). Both Llp1 and Llp2 were able to significantly reduce *S*. Typhimurium ATCC14028 biofilms at the highest tested concentration 200 μg/ml with ~ 90% for Llp2 and with ~50% for Llp1. Llp2 was still able to inhibit *S*. Typhimurium ATCC14028 biofilm at a concentration 10 μg/ml with significant decrease of 60%, but the inhibition was lost at concentration of 5 μg/ml. Llp1 did not show a significant inhibition below 50 μg/ml (Fig 2A). Therefore, 50 μg/ml was used for both of the lectins for further experiments to be able to compare the activities of Llp1 and Llp2. At a concentration of 50 μg/ml, the reduction in biofilm formation by *S*. Typhimurium ATCC14028 was on average with 40% for Llp1 and 90% for Llp2 (Fig 2B). Alternatively, the lectins were added after the adhesion phase (after 1.5 h) in which *S*. Typhimurium ATCC14028 was first grown on pegs without lectins allowing the bacterial cells to adhere. This resulted in a significant reduction in biofilm formation by *S*. Typhimurium ATCC14028, which was on average 20% for Llp1 and 92% for Llp2 at a concentration of 50 μg/ml (Fig 2B). No significant decrease in biofilm formation was observed when the lectin domains were added at the exponential growth phase after 8 hours or after 24 hours (Fig 2B). Nevertheless, when the lectin domains were added continuously, i.e. supplemented in the fresh medium added in the beginning and once after 24 hours, a significant reduction in the biofilm formation was also observed amounting to 70% for Llp1 and 93% for Llp2 (Fig 2B).

Absolute CFU counts of *S*. Typhimurium ATCC14028 biofilms grown in the presence of the lectin domains confirmed the antibiofilm activity of Llp1 and Llp2. In these experiments, Llp1 resulted in a reduction of biofilm growth by *S*. Typhimurium of 50% compared to the biofilm grown without lectins, while Llp2 resulted in 75% reduction (Fig 2C). Since in their natural context, the lectin domains are part of full length lectins, we also assessed the activity of the full length proteins, which exerted resulted in average reductions in biofilm formation of 79% and 77% for Llp1 and Llp2, respectively (Fig 2D). These inhibitions are similar to those observed for the corresponding lectin domains, though the full length Llp1 seems to be more active than its lectin domain alone.

Given the capacity of the lectins to prevent *S*. Typhimurium ATCC14028 biofilms, bioscreens were performed with the nutrient- poor medium (1/20 TSB) to provide the same growth conditions as during the biofilm experiments and to investigate whether the lectins also have an antimicrobial effect on growth in suspension. Interestingly, no inhibitory effect on planktonic growth was observed at concentrations of 200 μg/ml (Fig 2E). The growth of *S*. Typhimurium ATCC14028 was even increased when the lectin domains of Llp1 and Llp2 were added. However, this was not the case when *S*. Typhimurium ATCC14028 grown in nutrient-richer TSB medium (S1 Fig). These results suggest *S*. Typhimurium ATCC14028 is able to degrade Llp1 and Llp2 in poor medium and use them to grow, or lectins could promote sugar uptake.

Of note, ConA and HHA two well- known plant lectins were also included in the biofilm assay of *S*. Typhimurium ATCC14028 as a controls but no inhibition was observed when added at the 0 time point at concentration of 50 μg/ml (S1 Fig).

Since the lectins dramatically affected the biofilm formation of the model strain *S*. Typhimurium ATCC14028, other clinical *Salmonella* strains were included to investigate whether the lectin activity is strain- and species-specific (Fig 2F). Addition of the lectin domain of Llp2 resulted in a significant reduction (between 50% for SGSC2221 and 90% for most other strains) in the biofilm formation of all of the tested strains. In contrast, the lectin domain of Llp1 only inhibited the biofilm formation of three of the tested strains (SGSC2199, SGSC2221 and SGSC2459) (Fig 2F).

### Llp1 and Llp2 structurally disrupt *Salmonella* biofilms

**Fig 3:**
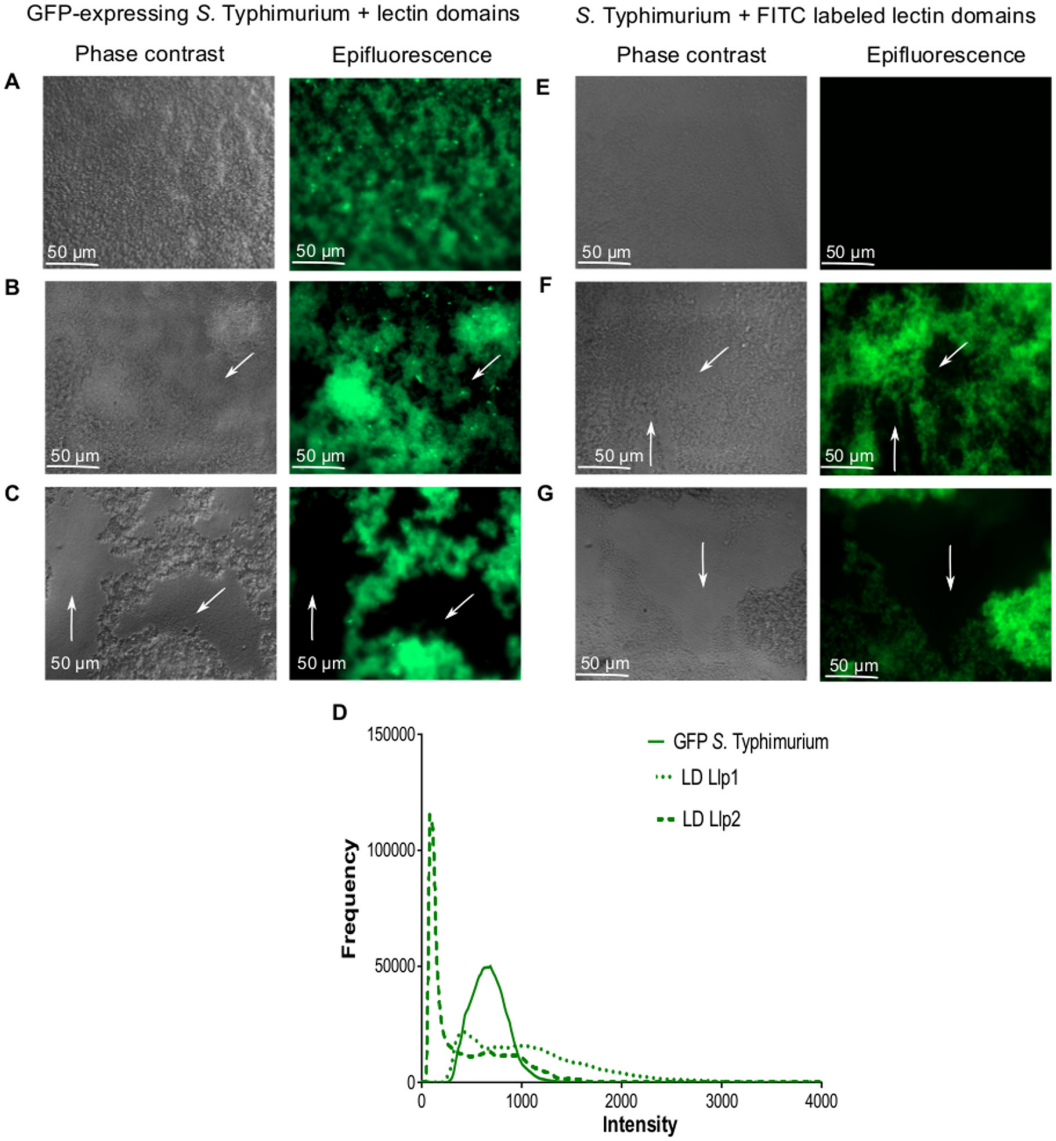
Biofilms of GFP-expressing *S*. Typhimurium ATCC14028 (A) or wild type *S*. Typhimurium ATCC14028 (E) grown in 1/20 TSB medium containing 50 μg/ml lectin domains of Llp1 (B) or FITC labeled Llp1 (F) and Llp2 (C) or FITC labeled Llp2 (G). Holes in the biofilm are indicated with arrows. (D) Fluorescent signal histogram of GFP-expressing *S*. Typhimurium ATCC14028 biofilm alone or treated with lectin domains of Llp1 and Llp2.

The divergent antibiofilm activity of Llp1 and Llp2 was also apparent when *S*. Typhimurium biofilms were visualized. As shown in Fig 3C, incubation in the presence of the lectin domain of Llp2 resulted in biofilms with large holes when compared to the negative control (Fig 3A). The biofilms treated with Llp1 appeared denser, but small holes were also observed (Fig 3B). In a second assay, biofilms of *S*. Typhimurium ATCC14028 were grown in the presence of FITC-labeled lectin domains. As shown in Fig 3F and 3G, both lectin domains appeared to cause the formation of holes, particularly visible even with a naked eye for Llp2. The results were also confirm by plotting the fluorescent frequency and intensity for each of the tested conditions for GFP-expressing *Salmonella* (Fig 3D). The fluorescent intensity of biofilms treated with Llp2 showed to be lower compared to the control (Fig 3D). The GFP-expressing *Salmonella* biofilms treated with Llp1 also showed lower fluorescence intensity as compared to the control, but with increased frequency.

### Llp1 and Llp2 have biofilm inhibition capacity beyond the gastrointestinal tract

**Fig 4:**
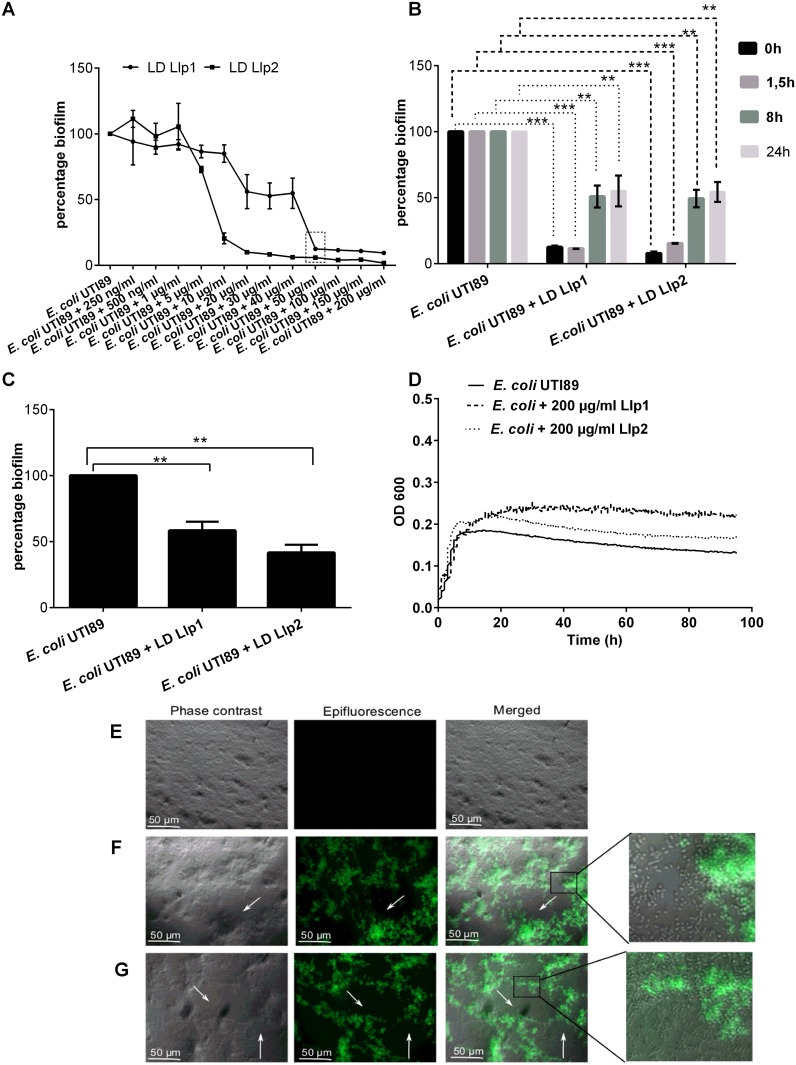
Effect of the lectin domains of Llp1 and Llp2 of *L*. *rhamnosus* GG on *E*. *coli* UTI89 biofilms. (A) Effect of the lectin domains (LD) of Llp1 and Llp2 on *E*. *coli* UTI89 biofilms added at different concentration at beginning of the biofilm formation. The lowest concentration in which both of the lectins showed significant reduction in the biofilm is indicated with a shaded bow. (B) The purified lectin domains of Llp1 and Llp2 were added after 0, 1.5, 8 and 24 hours to the biofilms. (C) Biofilm formation of *E*. *coli* UTI89 based on absolute cell counts. Biofilms were grown for 48h in 1/20 TSB without (control) for or with 50 μg/ml of lectin domain of Llp1 and Llp2. (D) Growth of *E*. *coli* UTI89 in the presence of lectin domain of Llp1 and Llp2 added at concentrations of 200 μg/ml. The error bars represent standard deviation of three independent experiments. The dataset comparisons are considered significant (p < 0.01 indicated with two asterisks or p< 0.001 indicated with three asterisks). (E) Biofilms of wild type *E*. *coli* UTI89 grown in 1/20 TSB medium containing 50 μg/ml FITC labeled Llp1 (F) and FITC labeled Llp2 (G). Holes in the biofilm are indicated with arrows. The zoomed images show single non-fluorescent bacterial cells, suggesting that lectins bind biofilm matrix and not the pathogenic cells.

As for *S*. Typhimurium ATCC14028, various biofilm assays were performed for the uropathogenic species *E*. *coli* UTI89 to investigate whether the lectins from *L*. *rhamnosus* GG can affect this pathogen’s biofilm formation. First, different concentrations of Llp1 and Llp2 were tested (Fig 4A). Similar to *S*. Typhimurium ATCC14028, both lectins showed an inhibitory activity at the highest tested concentrations 200 μg/ml with ~ 95% for Llp2 and with ~90% for Llp1 (Fig 4A). Llp2 was still able to inhibit *E*. *coli* UTI89 biofilm at a concentration of 10 μg/ml, with a significant decrease of 80%, but the inhibition was lost at concentration of 5 μg/ml. In comparison to *S*. Typhimurium ATCC14028, Llp1 showed a significant inhibition of the biofilms of *E*. *coli* UTI89 also at concentrations of 40 μg/ml, 30 μg/ml and 20μg/ml, but the inhibition was lost at 10 μg/ml. A concentration of 50 μg/ml was used for both of the lectins for further experiments. When purified lectin domains were added at a concentration of 50 μg/ml at the onset of biofilm development, Llp1 and Llp2 reduced the biofilm on average by 88 and 90%, respectively (Fig 4B). When the lectin domains were added after 1.5, 8 or 24h, both lectins could still reduce the biofilm formation as compared to the control, indicating that they can still inhibit later phases of biofilm development (Fig 4B). In agreement with the previous results, when determining the CFU counts, Llp1 and Lp2 caused significant reductions of *E*. *coli* UTI89 biofilm development, by on average 42% and 60% respectively (Fig 4C). Moreover, a bioscreen of *E*. *coli* UTI89 pointed out that the lectin domains did not affect the planktonic growth (Fig 4D), confirming that they have specific biofilm inhibitory activity without possessing anti-bacterial capacity. The activity of the well-known plant lectins ConA and HHA was also investigated as controls, but no inhibition was observed when added at the zero time point at concentration of 50 μg/ml (S1 Fig).

The *E*. *coli* UTI89 biofilms were also visualized to explore how the lectin domains structurally interfered with the biofilm formation. As shown in Fig 4F and 4G, incubation in the presence of the FITC-labeled lectin domain of Llp1 and Llp2 at a concentration 50 μg/ml resulted in biofilms with large holes when compared to the negative control (Fig 4E). Of interest, the FITC-labeled lectin domain of Llp1 and Llp2 were clearly distributed across the biofilm, but did not bind to the single cells (Fig 4F and 4G zoomed images).

### Llp1 and Llp2 show species-specific activity and increase biofilm formation of *Lactobacillus* species

**Fig 5:**
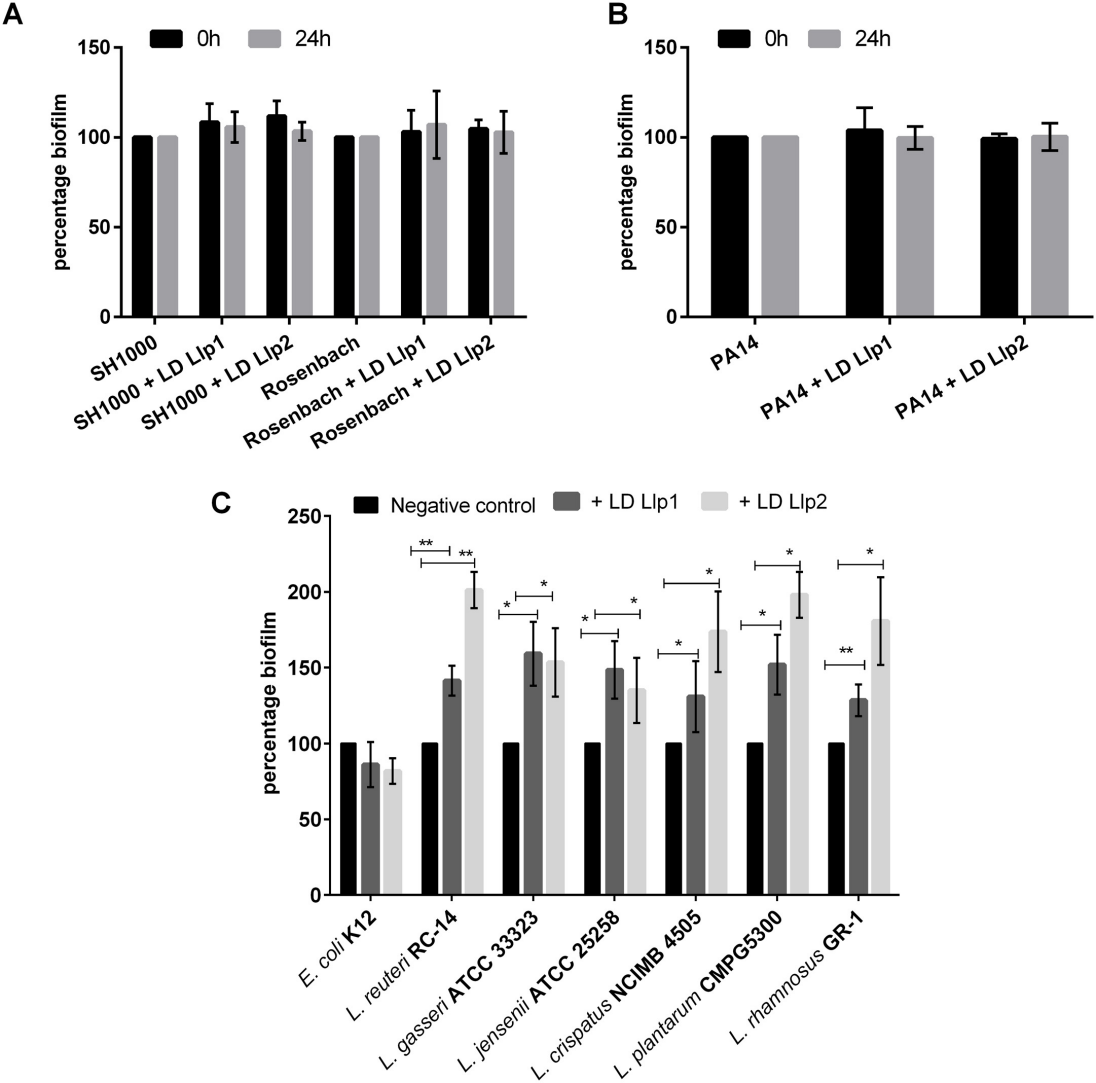
(A) Antibiofilm activity of the lectin domains of Llp1 and Llp2 of *L*. *rhamnosus* GG on *S*. *aureus* SH100 and Rosenbach added at zero time point and after 24 hours to the biofilms at a concentration of 200 μg/ml. (B) Antibiofilm activity of the lectin domains of Llp1 and Llp2 against *P*. *aeruginosa* PA14 added at zero time point and after 24 hours to the biofilm at a concentration of 200 μg/ml. (C) Antibiofilm activity of the lectin domains of Llp1 and Llp2 against beneficial members of the human microbiota. The error bars represent standard deviations of three independent experiments. The dataset comparisons are considered significant (p < 0.01 indicated with two asterisks or p< 0.001 indicated with three asterisks).

Since Llp1 and Llp2 can clearly inhibit the biofilm formation of *S*. Typhimurium ATCC14028 and *E*. *coli* UTI89, the activity against other bacterial species was also explored. Both Llp1 and Llp2 could not prevent biofilm formation of the other important pathogens *Staphylococcus aureus* SH1000 and Rosenbach (Fig 5A), nor of *Pseudomonas aeruginosa* PA14 (Fig 5B), suggesting that the Llp1 and Llp2 lectins have pathogenic species and strain-specific activity.

In addition, potential new anti-bacterial agents should not affect the beneficial bacteria of the human microbiota. Therefore, the activity of Llp1 and Llp2 against the biofilm formation of the beneficial *E*. *coli* K12 species and various *Lactobacillus* strains was also investigated. Of interest, biofilm formation of *E*. *coli* K12 was not significantly affected by Llp1 and Llp2 (Fig 5C), while the biofilm of the *Lactobacillus* strains was significantly increased with approximately 2 fold for most of the strains in the presence of Llp1 and Llp2 (Fig 5C).

### Llp1 and Llp2 show sugar specificity for complex glycans

**Fig 6:**
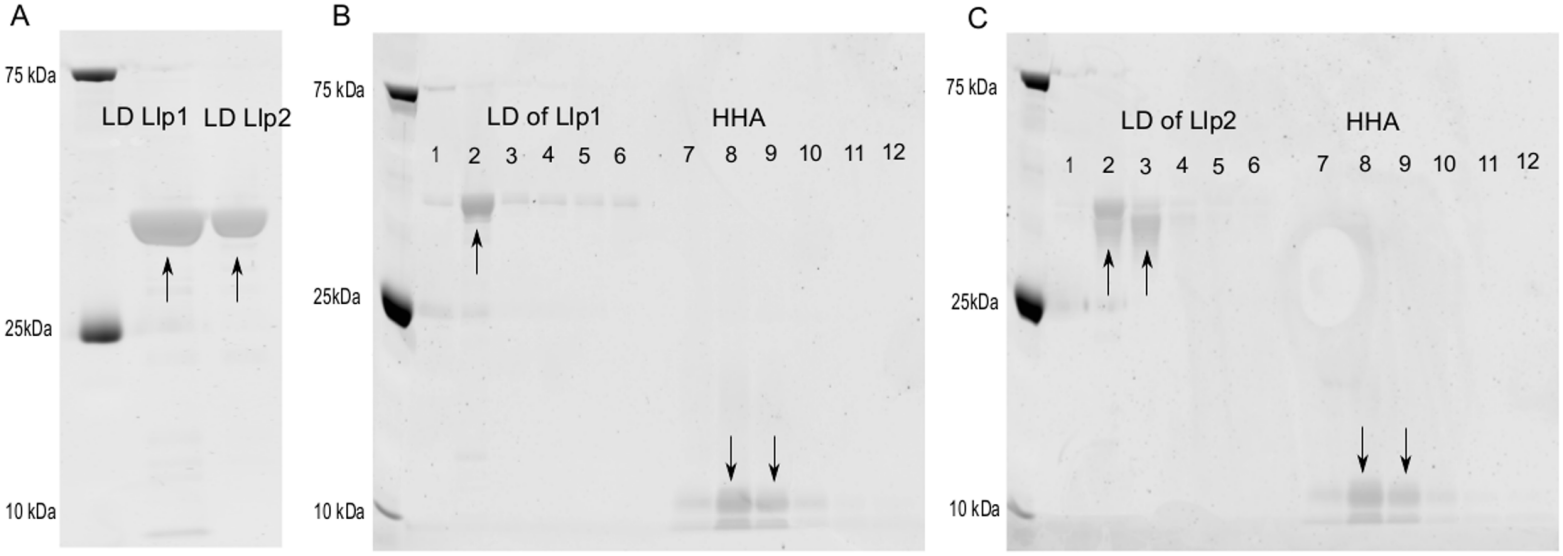
Determination of the sugar specificity of Llp1 and Llp2. (A) Purified lectin-like domains of Llp1 and Llp2 after size exclusion chromatography. (B) Determination of the sugar specificity of Llp1. (C) Determination of the sugar specificity of Llp2. Proteins that bound to sugar-coated Sepharose beads were separated by SDS-PAGE. Sepharose beads were coated with mannan (lane 2 and lane 8), D-mannose (lane 3 and lane 9), D-glucose (lane 4 and lane10), D-fucose (lanes 5 and lane 11), GluNAc (lane 6 and lane 12) or not coated with any sugar (lane 1 and lane 7, used as negative control). Arrows indicate the correct proteins bands. Each image represents a separate gel, which was run at different time points.

As an indication of the mode of action of Llp1 and Llp2, their sugar specificity was determined by pull-down sugar-binding assays using Sepharose beads coated with D-glucose, D-mannose, mannan, D-fucose and N-acetylglucosamine (GlcNAc) (Fig 6B and 6C). *Hippeastrum* hybrid lectin (HHA), a well-known mannose-specific plant lectin with molecular weight of 12.5 kDa, was used as positive control. The purified lectin domain of Llp1 showed the highest binding to mannan (Fig 6B, lane 2), whereas the lectin domain of Llp2 was binding to mannan and to D-mannose (Fig 6C, lane 2 and 3). For both lectin domains, there was no significant binding to any of the other sugars tested (glucose, fucose and GlcNAc).

**Fig 7:**
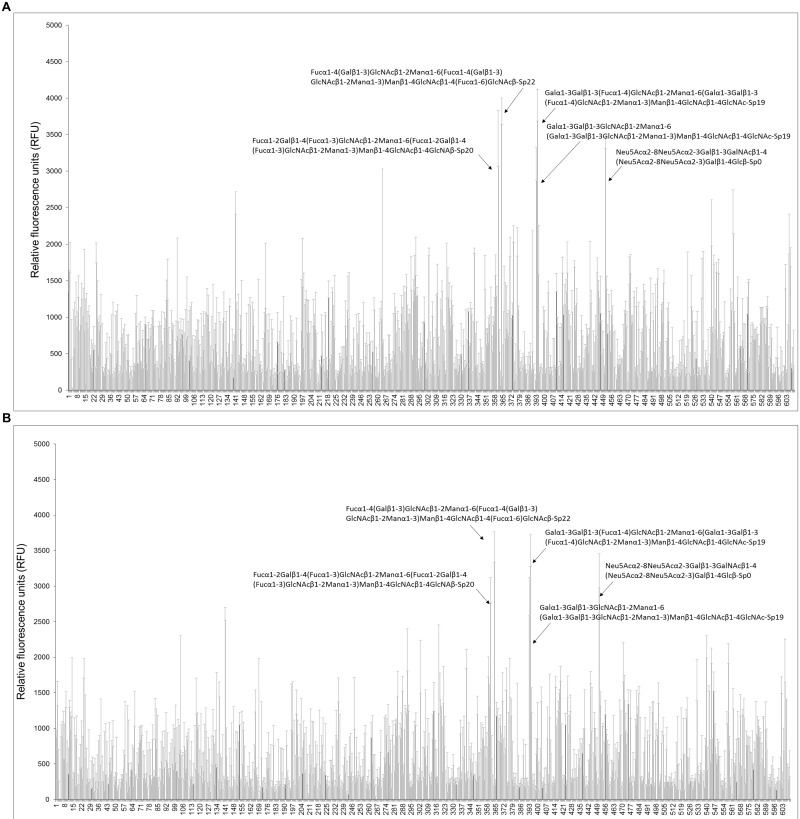
Glycan array used to determine the carbohydrate binding specificity of the lectin domain of Llp1 (A) and Llp2 (B). The glycan array was performed as described in Material and Methods. Sugars to which the FITC labeled lectin domains show the strongest binding are depicted.

Additionally, the purified FITC-labeled lectin domains were used for glycan array screening against a panel of more than 600 mammalian N-glycan structures. These data indicate that the lectin domains of Llp1 and Llp2 both recognize some complex N-glycans (Fig 7A and 7B), such as: i) Fucα1-4(Galβ1–3)GlcNAcβ1-2Manα1-6(Fucα1-4(Galβ1–3)GlcNAcβ1-2Manα1–3)Manα1-4GlcNAcβ1-4(Fucα1–6)GlcNAcβ-Sp22; ii) Galα1-3Galβ1-3(Fucα1–4)GlcNAcβ1-2Manα1-6(Galα1-3Galβ1-3(Fucα1–4)GlcNAcβ1-2Manα1–3)Manα1-4GlcNAcβ1-4GlcNAc-Sp19; iii) Fucα1-2Galβ1-4(Fucα1–3)GlcNAcβ1-2Manα1-6(Fucα1-2Galβ1-4(Fucα1–3)GlcNAcβ1-2Manα1–3)Manβ1-4GlcNAcβ1-4GlcNAβ-Sp20.

### Llp1 and Llp2 modulate the adhesion of *L*. *rhamnosus* GG to epithelial cells

**Fig 8:**
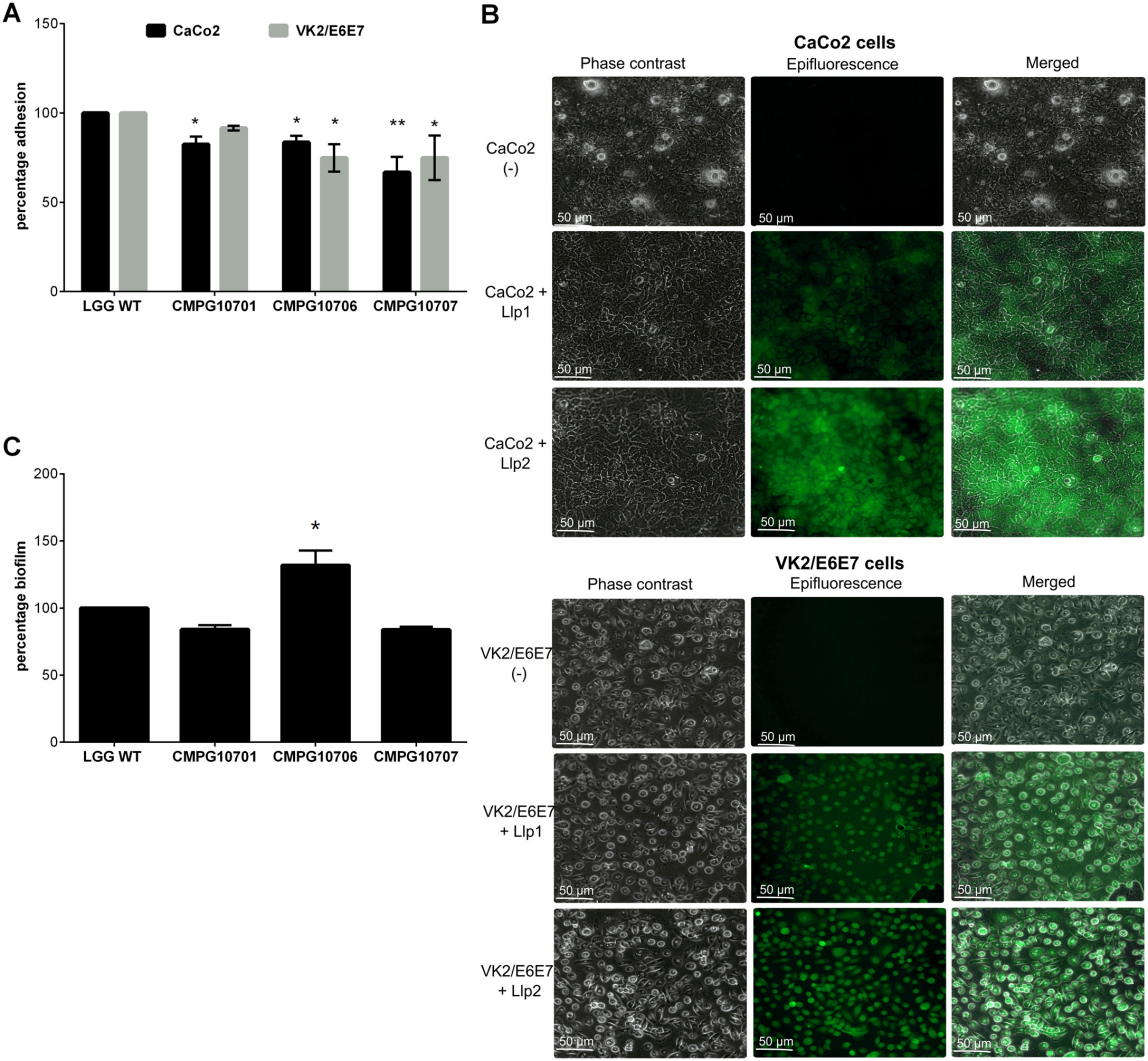
(A) Functional analysis of *llp1* (CMPG10701*)*, *llp2* (CMPG10706) and double (CMPG10707) mutant of *L*. *rhamnosus* GG for adhesion to gastrointestinal (CaCo2) and vaginal (VK2/E6E7) epithelial cells. The results are expressed relatively to the adhesion of *L*. *rhamnosus* GG wild-type, which was set at 100%. (B) Binding of FITC-labeled lectin domains of Llp1 and Llp2 to CaCo2 and VK2/E6E7 cells. (C) Functional analysis of *llp1* (CMPG10701*)*, *llp2* (CMPG10706) and double (CMPG10707) mutant of *L*. *rhamnosus* GG for biofilm formation. The error bars represent standard deviation of three independent experiments. The dataset comparisons (mutant pairwise to wild-type) are considered significant (p < 0.05 indicated with one asterisk in the picture or p < 0.01 indicated with two asterisks in the figure).

Since we envisaged that the *in situ* mucosal activity of the *Lactobacillus* lectins could be enhanced if they promote adhesion to the mucosa, we also studied the role of Llp1 and Llp2 in the adhesion capacity of *L*. *rhamnosus* GG cells. Hereto, we created corresponding knock-out mutants, including a double mutant of *llp1* and *llp2*. Functional analysis showed that CMPG10701 (*llp1* mutant) and CMPG10706 (*llp2* mutant) showed a minor but statistically significant reduction of 17% (p = 0.03) in adhesion to the intestinal epithelial cell line CaCo2. In contrast, CMPG10707 (double mutant in which both the *llp1* and *llp2* genes are knocked-out) showed a more pronounced reduction in adhesion by 34% (p < 0.01), suggesting a partially redundant role for Llp1 and Llp2 in adhesion to CaCo2 cells (Fig 8A). The complemented strains CMPG10702 and CMPG10715 showed complete restoration of the adhesion phenotype, while the double mutant in which only the *llp2* gene was re-introduced (CMPG10773) reached the same adhesion capacity as the *llp1* mutant CMPG10701 (S1 Fig). The role of Llp1 and Llp2 in the adhesion to vaginal epithelial cells VK2/E6E7 was also investigated. Only the CMPG10706 (*llp2* mutant) showed a significant reduced adhesion to VK2/E6E7 cells by 24% compared to the *L*. *rhamnosus* GG wild-type, suggesting a divergent role in adhesion to vaginal cells for the two lectins. Similarly, also biofilm formation assays suggest a different role for Llp1 and Llp2, with only the biofilm capacity of the *llp2* mutant CMPG10706 being altered compared to wild-type. In fact, this capacity was even increased by 30%, suggesting a suppressive role for Llp2 in biofilm formation (Fig 8C). However, studying the role of the lectins in the adhesion and biofilm capacity of *L*. *rhamnosus* GG is difficult in the context of the bacterial cells, because various other cell surface molecules may interfere and also have a role in adhesion. Therefore, the adhesion capacity of the purified FITC-labeled lectin domains of Llp1 and Llp2 after incubation with CaCo-2 and VK2/E6E7 cells grown on cover slips was also explored. Both lectin domains appeared to recognize and bind to the epithelial cells (Fig 8B).

## Discussion

In this study, we explored the role of two novel lectin-like proteins (Llp1 and Llp2) isolated from the probiotic *L*. *rhamnosus* GG and focused on their activity against various gastrointestinal and urogenital pathogens and beneficial species. Using a combined strategy of knock-out mutagenesis and heterologous expression of the lectin (domains) with advanced glycan specificity characterization assays and relevant bacterial functional characterization assays, we provide novel *in vitro* insights in the role of lectins in probiotic-pathogen-host cell interactions. To our knowledge, this is one of the first reports on the detailed characterization of a lectin-like proteins isolated from a probiotic strain.

L-type lectins are well documented and characterized in plants and animals [37,38], but not in bacterial species. However, the L-type lectin domain is part of several cell surface proteins of Gram-positive bacteria [39,40]. Interestingly Llp1 and Llp2 show only 35% sequence similarity at the protein level suggesting a possible gene duplication event followed by diversification. Of note, dynamic evolutionary diversification of genes encoding a L-type lectin receptor kinase (L-type LecRKs) and L-type lectin domain proteins (LLPs) has also been reported in plants [41]. The genes encoding L-type LecRKs and LLPs have been subject of strong positive selection, suggesting functional specialization in plant immunity. The differences observed in this work in the activity of Llp1 and Llp2 from *L*. *rhamnosus* GG, as further discussed below, suggest functional specialization of these lectins in lactobacilli.

Most intriguingly, we could show that the lectin domains of Llp1 and Llp2 can have a major impact on the biofilm development of multiple clinically relevant pathogens. First, both Llp1 and Llp2 lectin domains were able to prevent *S*. Typhimurium ATCC14208 biofilms, but with Llp2 showing the highest activity up to 90% reduction. This biofilm inhibition was only observed when the lectin domains were added at the onset or after 1.5 h of the biofilm formation and not on well-established biofilms. In contrast, both lectin domains were still significantly active against *E*. *coli* UTI89 even when added 24h after the onset of biofilm formation, indicating that they can also destroy established biofilms of this important uropathogenic pathogen. We even observed clear differences in biological activity between Llp1 and Llp2 when testing their activity against various clinical strains of *Salmonella*. Llp2 was active against most *Salmonella* species tested while the lectin domain of Llp1 only showed a much narrower spectrum. Thus, Llp1 and Llp2 clearly possess a different activity against different pathogens.

The localization of Llp1 and Llp2 within *Salmonella* and *E*. *coli* biofilms as observed after FITC-labeling suggests that they interact with components of the biofilm matrix. This matrix is composed of extracellular polymeric substances, including polysaccharides, proteins such as fimbriae and lectins, DNA and lipids [42] forming a cohesive network that plays an important role in stabilization of the biofilm, adherence of the bacterial cells to surfaces and cell interconnections. The biofilm matrix varies among strains, which may also explain the observed strain-specific and species-specific activity of the lectins against various clinical strains. For example, the biofilm matrix of *P*. *aeruginosa* contains the Psl and the Pel exopolysaccharides (rich in D-mannose, glucose and L-rhamosus) and alginate, while the biofilm matrix of *S*. *aureus* contains mainly poly-N-acetyl β-(1,6)-glucosamine [43,44]. However the biofilms of these species were not affected by Llp1 and Llp2. Both *S*. Typhimurium and *E*. *coli* biofilm matrices contain the polysaccharides cellulose (β-1,4-D-glucose polymer) and colanic acid (heteropolysaccharide of glucose, galactose, fucose and glucuronic acid) [45,46], which can be a target for Llp1 and Llp2 from *L*. *rhamnosus* GG. Of interest, the composition of the colanic acids closely resembles the composition of the complex N-type glycans to which Llp1 and Llp2 show specificity. Therefore, Llp1 and Llp2 might be able to bind to the glycosylated colanic acid and in this way destabilize the biofilm structure. This would explain the observed holes in the biofilms and the unstable biofilms formed by *S*. Typhimurium and *E*. *coli* strain after adding the lectins. In addition, cell surface-associated and extracellular lectins have been shown to play a role in the cross-linking of the polysaccharides and connecting the cells of pathogenic bacteria with the matrix of the biofilm. For example, outer membrane lectins LecA and LecB of *P*. *aeruginosa* are known to stabilize the biofilm [47,48], while aggregative fimbriae and proteins (BapA, Yeej and Bap) of *S*. Typhimurium and *E*. *coli* strains were demonstrated to stabilize the biofilm matrix via long-distance intercellular connections [49]. The exogenous added lectin domains from *L*. *rhamnosus* GG, as shown in this work, may therefore also compete with these crosslinking interactions and destabilize the biofilm. Of interest, Llp1 and Llp2 did not inhibit the biofilm formation of beneficial or commensal model species of the gastrointestinal and the vaginal environment. On the contrary, the Llp1 and Llp2 lectins were even found to increase the capacity of the *Lactobacillus* strains to form biofilms under the tested conditions.

To determine the sugar-dependent mode of action of the lectins, we aimed at characterizing their sugar specificity by using Sepharose beads binding assays and mammalian glycan array, which revealed specific binding of Llp1 and Llp2 to some complex N-glycan structures. This is in agreement with recent studies on plant lectins using glycan arrays showing that their specificity is manifold and cannot be merely described by single sugar monomers [50]. Plant L-type lectins have been reported as a family of lectins with diverse carbohydrate binding specificities, including mannose/glucose, galactose/GalNAc, GlcNAc, fucose and sialic acid. Interesting, the tested plant lectins HHA and ConA, showing overall mannose-specificity similar to Llp1 and Llp2, were not able to inhibit *S*. Typhimurium ATCC14208 and *E*. *coli* UIT89 biofilms. Therefore, the inhibitory effect of Llp1 and Llp2 on biofilm formation is probably based on binding to specific complex sugars in specific configurations. This is well in agreement with knowledge about plant lectins, that monomer specificity is insufficient to characterize specificity and avidity of lectins [50].

For bacterial lectins, only for a limited number of species, the sugar specificity has been determined, and glycan array analysis is rarely used. One important example of a well-characterized bacterial lectin is the soluble lectin from *P*. *aeruginosa* LecB (also known as PA-IIL). LecB binds to a large variety of fucosylated oligosaccharides, such as α-Fuc 1–2 Gal and β-Gal 1–4 α(Fuc1-3)GlcNAc, as confirmed with glycan array [51]. The sugar specificity of lectin-like adhesins of *E*. *coli*, present on the tip of pili or fimbriae, was also reported to include alpha-linked mannosides, galabiose (disaccharide of two galactose residues), sialylated galactose such as 2–3 sialyllactose and GalNac-β-1-4-Gal epitopes [52]. However, to the best of our knowledge, none of the studied bacterial lectins belong to the L-type lectins, which have –in contrast—been well characterized in plants and animals. Nevertheless, the exact sugar specificity of Llp1 versus Llp2 needs further exploration, since only mammalian glycans were screened with the glycan array. Importantly, bacterial glycans are generally far more diverse than eukaryotic glycoconjugates, as they can show an enormous diversity in monosaccharides building blocks, configuration, conformation and stereochemistry [53].

Finally, we could show that Llp1 and Llp2 play a modulatory role in the adhesion capacity of *L*. *rhamnosus* GG. It is well-known that SpaCBA pili are the key adhesins providing initial contacts, while other proteins play a secondary role [24,31], as shown in this work for Llp1 and Llp2. Of note, Caco2 cells were shown to have a rich glycosylation profile containing N-acetyl-D-lactosamine (LacNAc or Galβ(1,4)GlcNAc), Sia, GlcNAc glycoconjugates, branched mannosylated structures, as well as terminal αGal/αGalNAc-containing structures [54]. Based on the results of our biochemical analyses and glycan array screening, these sugar residues can form possible binding sites for Llp1 and Llp2. The role of lactobacilli lectins in host cell interaction is in agreement with the known function of lectins isolated from pathogenic bacteria such as *E*. *coli* and *P*. *aeruginosa*, which are important virulence factors mediating host-pathogen interaction [55]. However, the lectins from *L*. *rhamnosus* GG would then play a beneficial function by supporting the probiotic action of the strain and its adaptation to the gastrointestinal tract. The gastro-intestinal tract is the major site of application for *L*. *rhamnosus* GG, but the vagina has also been shown as (temporary) target site of *L*. *rhamnosus* GG [56]. In agreement, we also observed a role for the lectins in adhesion to vaginal epithelial cells. Hereby, mutant analysis suggests Llp2 has a more important role than Llp1 and is thus possibly being a vaginal niche-specific factor. Nevertheless, these roles need to be further explored *in vivo*.

In conclusion, the pronounced inhibiting effect of the isolated lectins on biofilm formation of common bacterial pathogens is worth to be explored in more detail and more complex models in further studies. Given the prevalence of problems associated with biofilms and the increased resistance of various bacteria against antibiotics, they probably hold the best potential for topical applications for pathogen exclusion, either alone or in combination with other antibacterials.

## Supporting Information

S1 Fig (A) Growth of *S*. Typhimurium ATCC14028 in presence of lectin domain of Llp1 and Llp2 (200 μg/ml) in TSB medium. (B) Effect of the plant lectins HHA and ConA on *E*. *coli* UTI89 and *S*. Typhimurium ATCC14028 biofilms added at the beginning of the biofilm formation at a concentration 50 μg/ml. The error bars represent standard deviations of three independent experiments. (C) Functional analysis for adhesion of CMPG10702, CMPG10715 and CMPG10773 to gastrointestinal CaCo2 epithelial cells. These are complementary mutants of the *llp1*, *llp2* and double mutant, respectively. The results are expressed relatively to the adhesion of *L*. *rhamnosus* GG wild-type, which was set at 100%. The error bars represent standard deviation of three independent experiments.
(TIFF)
